# Physical activity and affect in elementary school children's daily lives

**DOI:** 10.3389/fpsyg.2013.00456

**Published:** 2013-07-22

**Authors:** Jan Kühnhausen, Anja Leonhardt, Judith Dirk, Florian Schmiedek

**Affiliations:** ^1^Center for Research on Individual Development and Adaptive Education of Children at Risk (IDeA)Frankfurt, Germany; ^2^Center for Research on Education and Human Development, German Institute for International Educational Research (DIPF)Frankfurt, Germany; ^3^Department of Psychology and Sport Sciences, Goethe-UniversityFrankfurt, Germany

**Keywords:** physical activity, accelerometry, affect, children, daily life, FLUX, smartphone

## Abstract

A positive influence of physical activity (PA) on affect has been shown in numerous studies. However, this relationship has not yet been studied in the daily life of children. We present a part of the FLUX study that attempts to contribute to filling that gap. To this end, a proper way to measure PA and affect in the daily life of children is needed. In pre-studies of the FLUX study, we were able to show that affect can be measured in children with self-report items that are answered using smartphones. In the current article, we show that it is feasible to objectively measure children's PA with accelerometers for a period of several weeks and report descriptive information on the amount of activity of 51 children from 3rd and 4th grade. Additionally, we investigate the influence of daily PA on daily affect in children. Mixed effects models show no effect of PA on any of the four measured dimensions of affect. We discuss that this might be due to effects taking place at shorter time intervals, which can be investigated in future analyses.

## Introduction

Many people share the personal experience that physical activity (PA) can be an effective way to lift one's mood. Nevertheless, many people tend to lead a rather inactive, sedentary lifestyle. A recent large-scale survey among German children and youths revealed that only one out of about seven children meets the recommendation of the WHO to be physically active for at least 1 h per day (Krug et al., 2012). An association between PA and mental states has been confirmed in numerous studies. Physical inactivity has been shown to be associated with emotional problems (Kantomaa et al., 2008) and a higher risk for depressive and anxious mood in adolescents (Motl et al., 2004; Birkeland et al., 2009; Monshouwer et al., 2009; Jerstad et al., 2010; Rothon et al., 2010; Wiles et al., 2012). Accordingly, a longitudinal study indicated that PA can help to lift emotional problems in adolescent boys (Sagatun et al., 2007). Furthermore, not only the frequency but also the intensity of an activity has an effect on depressive and anxious affect with more vigorous activity being associated with reduced symptoms of depression and anxiety (Parfitt et al., 2009; Poole et al., 2011; Mata et al., 2012). In a recent meta-analysis, a beneficial effect of PA on mental health has also been shown for children (Ahn and Fedewa, 2011). Taken together, these studies support the notion that being physically active can yield an important contribution to psychological health and well-being. There are, however, some aspects that existing studies did not illuminate so far.

First, a major limitation of many studies is the focus on clinical samples and hence negative emotions. Oftentimes, studies investigate in how far PA can reduce symptoms of, for example, depression. How PA and affect are related in healthy subjects is studied less often. Second, many studies investigating the relationship between PA and affect are intervention studies. This means that subjects are required to do some sort of physical exercise, before and after which their affect is measured. If affect improves between the measurements, this effect is attributed to the exercise. In principle, this is a valid and sensible way to assess the influence of exercise on affect. The conclusions that can be drawn from it are, however, limited to conclusions about the influence of invoked exercise on affect. Whether everyday PA has an influence on everyday affect cannot be investigated (cf. Kanning et al., 2012). An approach that makes it possible to answer those two questions at once are intensive longitudinal studies. By measuring subjects repeatedly over a period of time, conclusions can be drawn about relationships between PA and affect within subjects and about the extent to which those effects differ between subjects. Furthermore, longitudinal studies are usually not designed as intervention studies, but rather measure habitual PA of subjects. Habitual PA must then, however, be reliably measured, which is not an issue in intervention studies. In many longitudinal studies, habitual PA is self-reported by the subjects. It has been shown that self-report measures of PA are not very reliable, especially when done retrospectively (e.g., Ebner-Priemer et al., 2006; Baumeister et al., 2007; Bussmann et al., 2009). In part, this is due to the fact that participants are more likely to answer questions regarding their PA, the more active they are (Dunton et al., 2012).

All of these problems have been stated before and attempts have been made to resolve them (e.g., Kanning and Schlicht, 2010; Kanning et al., 2012; Wichers et al., 2012). One successful approach was to let subjects fill out diaries right after self-induced activities over a prolonged period of time. This made it possible to assess effects within each subject and in their everyday lives. Results often showed that PA also had a positive effect on affect within single subjects (e.g., Kanning and Schlicht, 2010; Wichers et al., 2012). By not relying on retrospectively given information on PA, problems with self-report measures could presumably be reduced, but not completely avoided, in those studies. It is still left to the subject to judge what is considered PA. Clearly, it is desirable to measure PA without the need for self-reports from subjects. Advances in technological development make objective measures of PA a feasible alternative (for an overview of objective activity measurement techniques, see Bussmann et al., 2009; Bussmann and Ebner-Priemer, 2011; Butte et al., 2012).

Most studies fulfilling the above requirements—leaving the problems with subjective measurements of PA aside—are done with adults or adolescents (e.g., Kanning and Schlicht, 2010; Kanning et al., 2012; Wichers et al., 2012). Whether, and to what extent, relationships between PA and affect can be found in everyday life of children is not yet clear. We tried to close this gap, while at the same time avoiding the discussed methodological issues of previous studies. For that, our study needed to fulfill certain criteria. These demands are also stated and discussed in depth in the theoretical work by Kanning et al. (2013). First, we wanted to study the relationship between PA and affect in a group of healthy children. Second, these relationships should be investigated in the everyday life of children, without any intervention on PA. Third, we also wanted to investigate within-person effects. Thus, subjects had to be assessed repeatedly over a prolonged period of time. Finally, we wanted to measure PA objectively and not rely on self-report measures. As a result, bearing restrictions of generalizability in mind, our study should also yield information about the typical PA behavior of children in their everyday lives.

In sum, the current article has three main goals. The first goal is to establish the feasibility of measuring PA in the daily life of children over a period of several weeks. Obstacles to this are to be found on several levels. On the technical level, an objective method must be applied that can reliably measure children's PA. This method has to take specific demands of children's way of moving into account. For example, courses of movement tend to vary more between children than they do between adults, and children's activities are characterized by short bouts of movement (Bailey et al., 1995; Berman et al., 1998; Baquet et al., 2007; Corder et al., 2008). The second goal is to contribute to the description of PA of elementary school children in Germany. In order to do so, we consider average durations of PA per day, as well as differences in activity between children. The third goal is to investigate within-person relationships between children's PA and their affect on a day-to-day basis. Previous research indicates that the effect of PA on affect depends on the affect dimension used. For example, Wichers et al. (2012) found that PA positively influences positive affect, but does not influence negative affect. Therefore, to be able to thoroughly investigate the effect of PA on affect, we consider it very important to measure different dimensions of affect. To the best of our knowledge, to date there is no comparable study with children that would allow to formulate specific expectations about which affect dimensions PA would influence in children. Therefore, our investigation has to be considered exploratory.

## Materials and methods

### Flux study

The current study was part of the FLUX (Assessment of Cognitive Performance FLUctuations in the School ConteXt) study, which aims at investigating daily fluctuations in children's cognitive performance in the school context. One-hundred and ten children received smartphones on which they worked on working memory tasks and answered self-report questionnaires several times a day for 4 weeks. Additionally, 82 of these children received accelerometers to wear for the time of the study. This way, mutual relationships between working memory performance, self-report measures such as affect and motivation, and PA can be assessed in real life conditions. The study was approved by the ethics committee of the Goethe-University in Frankfurt, Germany. For their participation, subjects received a reward in the form of money or a gift coupon.

### Sub-sample

In the beginning of the FLUX study, a sample of 82 children (45% girls) received accelerometers to measure their PA. The children were in third or fourth grade, with their age ranging from 97 to 132 months (*M* = 117.2, *SD* = 7.4 months). In total, there were three third grade and three fourth grade classes. The average size of the classes was 22.3 (*SD* = 1.4). On average, the children weighed 34.4 kg (*SD* = 6.5 kg), were 139.7 cm tall (*SD* = 6.5 cm) and had an average *Body-Mass-Index* (BMI) of 17.5 (*SD* = 2.6).

### Measuring physical activity

#### Accelerometer

Acceleration was measured with the ActiGraph GT3X+ (ActiGraph, LLC, Fort Walton Beach, FL). The GT3X+ is a triaxial accelerometer that measures acceleration in a range from −6 to +6 g. As it is usual for children, the devices were worn on the waist (Strath et al., 2012). The sampling rate was set to 30 Hz. This sampling rate is sufficiently precise for measuring PA while not producing too much data to be stored on the devices when recording for more than a week.

#### Reference measurements

Acceleration data were analyzed using *reference-pattern-based classification* (e.g., Foerster and Fahrenberg, 2000). This method is based on the idea that each individual moves in a unique way. This means that the same activity will produce different acceleration data when done by different persons. To correctly classify individual acceleration data into activity categories, individual datasets are required for which it is exactly known which acceleration data corresponds to which activity. Such datasets can be acquired in reference measurements, based on a pre-defined protocol of activities. They can then be used to categorize data as representing a certain activity. In the present study, reference measurements were conducted in classes of children in a single physical education lesson per class. The conducted activities are specified in Table 1. To take into account that the children may not always wear their accelerometer, data that was recorded when the device was not worn was also included in the analyses. For that, a recording accelerometer was placed on a table in three different positions. The positions were the ones we identified as most likely for a device lying on a flat surface, due to the shape of the device. The resulting raw acceleration data, measured in g, were summarized in a set of key-values in non-overlapping time frames. These key-values were the mean acceleration value of the frame, the corresponding variance, the correlations between the acceleration values of the three axes, and the energy (see Bao and Intille, 2004). To account for the fact that the movement of children tends to be irregular and characterized by short bouts of vigorous movement (e.g., Baquet et al., 2007; Corder et al., 2008) we chose a relatively short frame length of 2.5 s. With the chosen sampling rate of 30 Hz this means that each frame consisted of 75 acceleration measurements on each of the three axes. With acceleration being measured on three axes, this leads to 12 key-values describing each frame.

**Table 1:** **The six activities conducted in the reference measurements, classified as sedentary behavior, inactive behavior, or physical activity (moderate and vigorous)**.

Sedentary behavior	Lying comfortably
	Sitting upright
Inactive behavior	Standing upright
Physical activity	Walking at a normal pace
	Walking at a fast pace
	Running

The resulting data were classified using *support vector machines* (SVMs), which have proven to be very effective for classification problems (e.g., He and Jin, 2009). The SVM approach was first introduced by Boser et al. (1992) and further developed to what is used in the present study by Cortes and Vapnik (1995). A detailed description of SVMs is beyond the scope of this article. Interested readers who want to delve deeper into this topic are referred to Cristianini and Shawe-Taylor (2000). A rather detailed user's guide that contains theoretical background as well as practical advice for the usage of SVMs is provided by Ben-Hur and Weston (2010). We conducted SVMs with the function “svm” from the package “e1071” (Dimitriadou et al., 2011) of the open-source statistical software R (R Development Core Team, 2012). Regarding the specific settings of the function, we followed the recommendations of the author's manual (Meyer, 2012). The results of the classification process are reported elsewhere (Kühnhausen et al., in preparation). There, we show that our method enabled us to correctly classify activities with great precision. Thus, the trained SVM models were used to classify the acceleration data collected during the 4 week study phase. For those children who were present during the reference measurement, an individual model, only trained on the data of the respective child, was used. For the children who were not present, a general model, based on the reference data of the children who did take part in the reference measurements, was used.

#### Measures of PA

After cleaning the data (see below), they were collapsed into standard measures of PA. Lying and sitting were classified as *sedentary behavior*. Standing was classified as *inactive behavior*. Slow and fast walking were classified as *moderate to vigorous physical activity* (MVPA). Running was classified as *very vigorous PA*. PA was then defined as the percentage of active time per day (MVPA plus *very vigorous activity*). Due to the finding that not only the total amount of PA, but also the amount of very vigorous PA, may have an influence on affect (Parfitt et al., 2009; Poole et al., 2011; Mata et al., 2012) the percentage of the active time that was spent with *very vigorous activity* was also included as a predictor of affect. The distinction between sedentary and inactive behavior is theoretically important. However, it is not relevant for our current analyses, since we only investigate how being physically active influences affect.

#### Data cleaning

The resulting activity data was further processed to dispose of spurious data. In a first step, consecutive strings of 1 h of classified *non-wear time* were identified. Within this—at least—1 h of *non-wear time*, 5 min of activity were allowed. Thus, a maximum of 5 min of activity, bordered by at least an hour of *non-wear time*, was identified as spurious activity and re-classified as *non-wear time*. Shorter periods of *non-wear time* were considered very steady phases of *sedentary behavior* and thus re-classified as just that. The second step was to restrict the data to adequate wear times. For this, the self-reported information about sleeping behavior was used. Each morning the children had to report when they went to bed the night before and when they woke up in the morning. This information was used to identify wake time of each child on each day. In further analyses only these time intervals were considered. In a third step, days of data were checked and only valid days remained for further analyses. A valid day was defined as a day with at least 6 h of activity data, that is, a day on which the device was worn for at least 6 h.

### Measuring affect

Affect was measured four times a day with 12 items that have proven to reliably measure affect in children. Each item contained a statement, for example “Right now I feel content”, that had to be rated on a 5-point Likert scale, ranging from 1 (“not at all”) to 5 (“very much”). The 12 items were selected out of a large item pool in a pre-study of the FLUX project (Leonhardt et al., in preparation). A detailed description of these items and their psychometric properties can be found there. In short, the items were selected for their ability to measure affect structures in groups of children, as well as within children over time. Factor analyses showed that the latent affect factors *pleasantness, unpleasantness, activation*, and *deactivation* can be represented with three items each and be used to describe affect differences between persons as well as fluctuations in affect within persons over time. The within-person reliability scores of all factors were above 0.59 (for details, see Leonhardt et al., in preparation). For the current study, composite scores were created for each triplet of items belonging to one of these four factors. These scores were averaged across the four measurement occasions for each day. This meant that for each child, one score per day could be obtained for each measured dimension of affect.

### Analyses

The influence of daily PA on daily affect was analyzed with mixed effects models (also called hierarchical linear models, e.g., Raudenbush and Bryk, 2002). A linear trend over time was included in all models. Separate models were calculated for the influence of both PA variables on each affect dimension. The corresponding regression equations are given by:

$$\begin{array}{l} \text{Level }1:{\text{Affect}}_{ti}=\beta_{0i}+\beta_{1i}\;{\text{Trend}}_{ti}+\beta_{2i}\;{\text{PA}}_{i}+\beta_{3i}\;{\text{PA}}_{ti}+\varepsilon_{ti}\\ \text{Level }2:\beta_{0i}=\gamma_{00}+\sigma_{0i}\\ \;\beta_{1i}=\gamma_{10}+\sigma_{1i}\\ \;\beta_{2i}=\gamma_{20}\\ \;\beta_{3i}=\gamma_{30}+\sigma_{3i} \end{array}$$

The first level represents the different measurement occasions (i.e., days) within each child. On this level, within-person effects are modeled. Children's daily affect is predicted by a general intercept (β_0*i*_), a linear trend over time (β_1*i*_), the average PA of each child over the time of the study (β_2*i*_), the deviation of each child from its own average PA (β_3*i*_), and a residual term (ε_*ti*_). The subscript *i* refers to the different children, the subscript *t* refers to different measurements of each child. Effects on this level can be interpreted as average effects across all children (fixed effects). The second level represents the level of the different children. On this level, between-person differences in the effects on the first level are accounted for (random effects). The above model equations describe the final model. To assess the relevance of the effects, all corresponding reference models were also fitted to the data for all affect dimensions and PA variables. Thus, first an empty model was obtained. Next, the fixed effects were added to the model. Finally, the random effects were added resulting in the final model. The covariance of the random intercept and random trend was also included in the model. Since this covariance does not play a substantial role for the interpretation of the models, it will not be reported in the Results section. All models were calculated with *restricted maximum likelihood* (REML) estimation using SAS PROC MIXED (SAS Institute, Inc., Cary, North Carolina).

## Results

### Dropout and valid data

Of the 82 children who received an Actigraph in the beginning, 13 returned their device in the course of the study. Five children lost their Actigraph, and one device was stolen. Two devices turned out to have technical faults, resulting in almost constantly recording the maximum acceleration. This leaves 61 children whose data could in principle be used. Of those children, 10 did not wear their device regularly, leading to less than 4 days of valid data. These children were also excluded from further analyses. This left 51 children with sufficient data to reliably describe their typical activity behavior.

### Compliance

**Figure 1:**
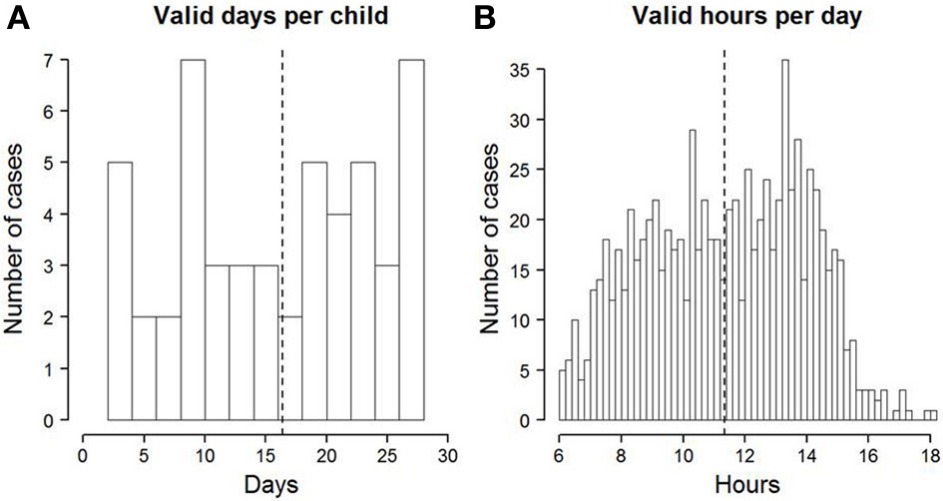
**(A)** Histogram of the number of valid days per child. The dotted line depicts the mean across all children. **(B)** Histogram of the number of valid hours per day. Days with less than 6 valid hours were excluded from analyses. The dotted line depicts the mean across all days and children.

Overall, compliance among the children was satisfying. On average, the 51 children whose data were analyzed had almost 17 valid days of data (*SD* = 8; see also Figure 1A). The distribution of the number of valid days has a slightly bi-modal shape. There are several children who have more than 20 days of data. At the same time, a considerable group of children have only ten or less days of data. This is in line with our observation that the compliance differed considerably between children. A satisfying compliance can also be asserted from the average time the devices were worn per day (see Figure 1B). On an average valid day, the devices were worn for almost eleven and a half hours (*SD* = 2.6). On the majority of days, the devices were worn between 8 and 15 h. It thus seems that those children who were willing to wear their device until the end of the study did so with good compliance.

### Descriptives

**Figure 2:**
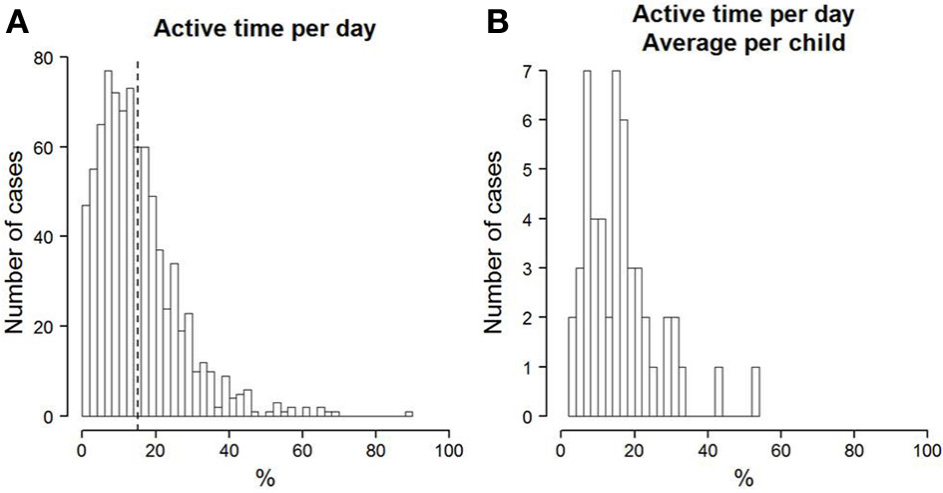
**(A)** Histogram of the percentage of time per day that children were physically active. The dotted line depicts the mean across all days and children. **(B)** Histogram of each child's mean percentage of time per day spent with PA.

**Figure 3:**
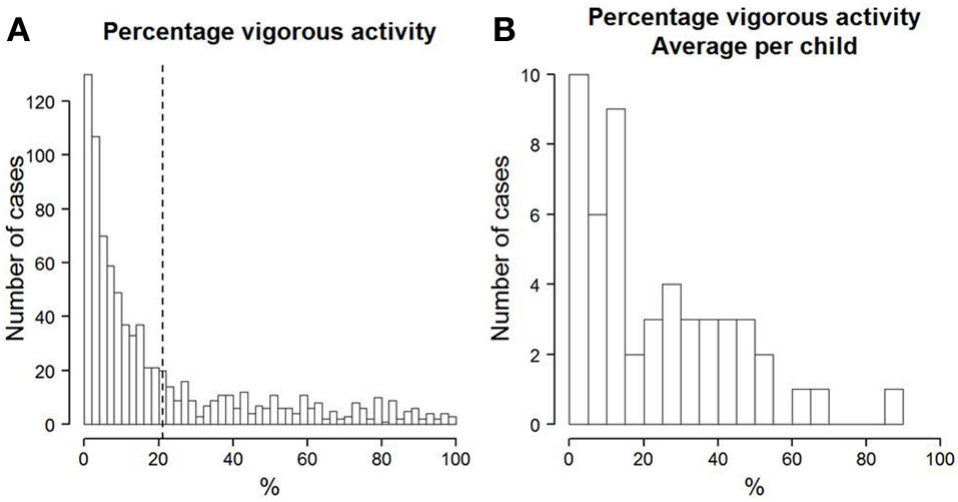
**(A)** Histogram of the percentage of active time per day that children spent with very vigorous PA. The dotted line depicts the mean across all days and children. **(B)** Histogram of each child's mean percentage of the active time per day that was spent with vigorous PA.

On an average day, the children were active for about 15% of the time. This corresponds to about 103 min of activity (*SD* = 81). The active time differed considerably between days, as can be inferred from the rather large standard deviation (see also Figure 2A). The average active time per day for each child (see Figure 2B) shows considerable differences between children. It is clear that there are some children who are generally more active than others. A similar picture can be seen in the time that is spent in very vigorous activity. On average, of the total active time, about 21% was spent in very vigorous activity (see Figure 3A). This corresponds to an average of about 21 min of vigorous activity per day (*SD* = 24). Just as the overall activity, the amount of vigorous activity also varied considerably between children (see Figure 3B). A two-level multi-group model with maximum-likelihood (MLR, Mplus 7.0, see Muthén and Muthén, 1998–2012) estimation showed no significant difference between boys and girls in the total amount of active time per day. Boys did, however, show a higher percentage of vigorous activity than girls (*M*_difference_ = 0.11, *SE* = 0.05, *p* < 0.05). Neither the total activity per day, nor the percentage of vigorous activity correlated significantly with children's BMI. The intra-class-correlation (*ICC*) of the measures of total activity was 0.23. The *ICC* of the percentage of very vigorous activity was 0.29. The *ICC* relates the variance that is found between children to the total variance. Thus, one minus the *ICC* is the part of the total variance that can be assigned to within-subject variation. The resulting *ICC* values indicate that there were substantial within-subject fluctuations in the measures of PA.

The results of the affect measurements are depicted in Table 2. Overall, children rated their affect relatively high on the more positive affect dimensions (*pleasantness* and *activation*) and relatively low on the more negative dimensions (*unpleasantness* and *deactivation*). The average intra-individual standard deviation (ISD) is the mean of each child's deviation from his or her own average value on the respective affect factor. The resulting values did show that, on average, children's affect varied considerably across days. The *ICC* values of the affect factors also indicate that there were substantial affect fluctuations.

**Table 2:** **Descriptive statistics of the four factors of affect (measured on a five-point Likert-scale from 1 to 5)**.

	**Mean *(SD)***	**Average ISD**	**ICC**
Pleasantness	3.95 (1.00)	0.55	0.51
Unpleasantness	1.46 (0.73)	0.43	0.52
Activation	3.60 (1.19)	0.62	0.59
Deactivation	1.62 (0.78)	0.49	0.44

ICC, intra-class-correlation; ISD, intra-individual standard deviation.

### Models

The results of all fitted models are depicted in Table 3. Overall, there were no reliable effects of daily PA on daily affect. This was the case for the effect of overall PA, as well as for vigorous PA on the four measured factors of affect.

**Table 3:** **Parameter estimates of the described models (standard errors in parentheses)**.

	**Empty model**	**Trend only**	**Total activity: fixed effects only**	**Total activity: fixed and random effects**	**Vigorous activity: fixed effects only**	**Vigorous activity: fixed and random effects**
**PLEASANTNESS**						
Fixed intercept	3.937^*^ (0.11)	4.122^*^ (0.10)	3.982^*^ (0.19)	3.982^*^ (0.19)	4.006^*^ (0.15)	4.006^*^ (0.15)
Trend (time)		−0.014^*^ (0.005)	−0.012^*^ (0.006)	−0.012^*^ (0.006)	−0.011^*^ (0.006)	−0.011^*^ (0.006)
Average activity			0.970 (1.02)	0.970 (1.02)	0.569 (0.51)	0.569 (0.51)
Daily activity			0.054 (0.29)	0.054 (0.29)	−0.056 (0.13)	−0.056 (0.13)
Random intercept	0.63^*^ (0.13)	0.438^*^ (0.10)	0.380^*^ (0.10)	0.380^*^ (0.10)	0.368^*^ (0.10)	0.368^*^ (0.10)
Variance daily activity				0.00 (NA)		0.00 (NA)
Variance trend		0.0009^*^ (0.0002)	0.0009^*^ (0.0002)	0.0009^*^ (0.0002)	0.0009^*^ (0.0002)	0.0009^*^ (0.0002)
Residual variance of pleasantness	0.409^*^ (0.02)	0.324^*^ (0.01)	0.297^*^ (0.02)	0.297^*^ (0.02)	0.297^*^ (0.02)	0.297^*^ (0.02)
−2 Log-Likelihood	2263.6	2095.8	1345.6	1345.6	1348.1	1348.1
**UNPLEASANTNESS**						
Fixed intercept	1.505^*^ (0.08)	1.435^*^ (0.07)	1.530^*^ (0.15)	1.530^*^ (0.15)	1.507^*^ (0.12)	1.508^*^ (0.12)
Trend (time)		0.006^*^ (0.002)	0.008 (0.005)	0.008 (0.005)	0.008 (0.005)	0.008 (0.005)
Average activity			−0.855 (0.80)	−0.855 (0.80)	−0.525 (0.40)	−0.523 (0.40)
Daily activity			−0.231 (0.22)	−0.231 (0.25)	−0.125 (0.10)	−0.074 (0.16)
Random intercept	0.354^*^ (0.07)	0.241^*^ (0.06)	0.233^*^ (0.06)	0.233^*^ (0.06)	0.228^*^ (0.06)	0.225^*^ (0.05)
Variance daily activity				0.229 (0.49)		0.345 (0.28)
Variance trend		0.0002^*^ (0.00008)	0.0007^*^ (0.0003)	0.0007^*^ (0.0003)	0.0007^*^ (0.0003)	0.0008^*^ (0.0003)
Residual variance of unpleasantness	0.205^*^ (0.009)	0.187^*^ (0.009)	0.157^*^ (0.009)	0.157^*^ (0.009)	0.157^*^ (0.009)	0.150^*^ (0.009)
−2 Log-Likelihood	1512.9	1462.9	910.7	910.7	912.6	909.1
**ACTIVATION**						
Fixed intercept	3.604^*^ (0.14)	3.857^*^ (0.13)	3.977^*^ (0.26)	3.977^*^ (0.26)	3.847^*^ (0.21)	3.847^*^ (0.21)
Trend (time)		−0.020^*^ (0.006)	−0.018^*^ (0.006)	−0.018^*^ (0.006)	−0.018^*^ (0.006)	−0.017^*^ (0.006)
Average activity			−0.552 (1.36)	−0.552 (1.36)	0.154 (0.69)	0.157 (0.69)
Daily activity			0.112 (0.29)	0.112 (0.29)	−0.109 (0.13)	−0.142 (0.15)
Random intercept	0.905^*^ (0.19)	0.778^*^ (0.17)	0.826^*^ (0.19)	0.826^*^ (0.19)	0.807^*^ (0.19)	0.805^*^ (0.19)
Variance daily activity				0.00 (NA)		0.089 (0.16)
Variance trend		0.001^*^ (0.0003)	0.001^*^ (0.0003)	0.001^*^ (0.0003)	0.001^*^ (0.0003)	0.001^*^ (0.0003)
Residual variance of activation	0.456^*^ (0.02)	0.340^*^ (0.02)	0.274^*^ (0.02)	0.274^*^ (0.02)	0.274^*^ (0.02)	0.272^*^ (0.02)
−2 Log-Likelihood	2329.1	2121.5	1297.4	1297.4	1299.5	1299.5
**DEACTIVATION**						
Fixed intercept	1.662^*^ (0.08)	1.707^*^ (0.09)	1.738^*^ (0.17)	1.738^*^ (0.17)	1.818^*^ (0.13)	1.819^*^ (0.13)
Trend (time)		−0.003 (0.003)	−0.002 (0.005)	−0.002 (0.005)	−0.002 (0.005)	−0.002 (0.005)
Average activity			−0.640 (0.87)	−0.640 (0.87)	−0.806 (0.42)	−0.809 (0.42)
Daily activity			−0.258 (0.26)	−0.258 (0.26)	0.023 (0.11)	0.034 (0.15)
Random intercept	0.347^*^ (0.07)	0.341^*^ (0.08)	0.321^*^ (0.08)	0.321^*^ (0.08)	0.284^*^ (0.07)	0.284^*^ (0.07)
Variance daily activity				0.00 (NA)		0.054 (0.22)
Variance trend		0.0005^*^ (0.0002)	0.0008^*^ (0.0002)	0.0008^*^ (0.0002)	0.0008^*^ (0.0002)	0.0008^*^ (0.0002)
Residual variance of deactivation	0.280^*^ (0.01)	0.247^*^ (0.01)	0.214^*^ (0.01)	0.214^*^ (0.01)	0.214^*^ (0.01)	0.212^*^ (0.01)
−2 Log-Likelihood	1833.7	1775.3	1107.4	1107.4	1108.4	1108.3

Model results are displayed separately for each measured factor of affect.

* p < 0.05.

## Discussion

We were able to demonstrate the feasibility of objectively measuring PA in the daily life of children over a period of several weeks. There was a considerable group of children who were willing to wear their devices during most of the days of our study and during most of the waking hours of each day. We hope that this can increase the motivation of researchers to conduct similar studies and further contribute to this area of research. By successfully measuring PA in a group of children over a prolonged period of time, we were also able to describe typical PA behavior in those children. This contributes to a more complete picture of typical PA in everyday life of elementary school children in Germany.

We did not find a significant influence of daily PA on any of the four affect factors. There are several possible explanations for that. The most apparent one is a lack of power. The numerical decrease of the residual variance of some of the affect variables when including the fixed and random effects of PA in the model suggests that effects might exist. For example, consider the effect of PA on the affect dimension *activation*. The residual variance of *activation* reduces from 0.340 in the model that only includes the trend, to 0.274 when also including the total active time per day. Thus, including PA to predict *activation* reduces its residual variance by over 19%. However, all effects fail to be statistically significant. This might be attributed to the fact that habitual PA does not show very great variability. To some extent, this might be due to the way we processed the acceleration data and classified it into certain activities. A lack in variability may result in a reduced likelihood of detecting reliable effects. It could therefore well be that with larger samples of children and/or occasions, statistically significant effects could be found. This assertion is supported by the conclusion in the meta-analysis of Ahn and Fedewa (2011) that “effects of PA on mental health were small but significant”. By all means, the results are no reason to exclude the possibility that daily PA may have an effect on daily affect in children. This assertion leads to a second possible explanation for the non-significance of the results. It is quite possible that the influence of PA on affect is different for different children. In the literature, this suggestion is supported by the finding that the influence of PA on positive affect in adults depends on the baseline level of positive affect (Kanning and Schlicht, 2010). In the present study, this may be indicated by the fact that adding the random effects of PA on affect to the model slightly decreased the residual variance of some of the affect dimensions. It might be worthwhile to more closely analyze the multivariate time series of single participants to see if for some of them reliable influences of PA on affect can be detected. Finding those effects in some but not all children could also partly explain the non-significance of the results. For the whole group, the effects would just not be strong enough to reach significance. Finally, and maybe most importantly, reasons for the non-significance of the effects might be found in the design of the analyses. All studies that have found an effect of PA on affect have one thing in common. Affect was measured directly after being physically active. Even those studies that measure subjects in their daily lives (e.g., Kanning and Schlicht, 2010; Wichers et al., 2012) let their subjects rate their affect right after a self-reported period of PA. A detailed description of conceptual advantages as well as methodological considerations of an improved version of this method, *interactive ambulatory assessment*, is given by Ebner-Priemer et al. (2013). Most importantly, it enables researchers to measure affect right after an accelerometer has detected that a subject was physically active. This greatly enhances the likelihood to detect an influence of PA on affect (Ebner-Priemer et al., 2013). In the study by Wichers et al. (2012), the positive influence of PA on affect was only present in the 180 min after the activity. Furthermore, Bossmann et al. (2013) found a positive influence on subject's mood by the intensity of the PA that was conducted in the 10 min before the affect measurements. In the present article, we calculated the average affect during the whole day and related it to the PA during the whole day. It is conceivable that this is a reason for the absence of significant effects (cf. Schwerdtfeger et al., 2008). Following this argument, our next steps will include the investigation of the influence of PA on affect on different time scales. Since we measured PA continuously during the whole day and affect four times a day, our data allows for quite some flexibility in this respect. One approach will be to only look at the PA that immediately preceded the affect measurements. The usefulness of this approach is suggested by the discussed empirical findings. In addition, there is the theoretical consideration that “we might expect that different [time] intervals will yield different patterns of variability and result from different influences on the ‘system’ or individual” (Martin and Hofer, 2004, p. 10).

Studying the relationship between PA and affect can be done in many different ways. Our results indicate that the design of a study, especially with regards to the investigated time-scale, can have an influence on the results. By further exploring sensitive time intervals for the effects of PA on affect, our study may help researchers to choose a proper study design. Furthermore, we were able to show that objective measures of PA are by now feasible, even in prolonged longitudinal studies with children. Intensive longitudinal studies that use ambulatory assessment also allow for the investigation of effects in a way that cross-sectional research designs cannot provide. For example, considering the problems that can be caused by inactivity, it is desirable to investigate possible reasons for people being inactive. Intensive longitudinal studies can clarify these reasons and thus give hints on how to help people to become more active (cf. Ebner-Priemer et al., 2013). Generally speaking, our study shows the great opportunities available for future researchers who want to study PA in children.

### Conflict of interest statement

The authors declare that the research was conducted in the absence of any commercial or financial relationships that could be construed as a potential conflict of interest.
